# Applying the Innov8 approach for reviewing national health programmes to leave no one behind: lessons learnt from Indonesia

**DOI:** 10.1080/16549716.2018.1423744

**Published:** 2018-03-23

**Authors:** Theadora Swift Koller, Victoria Saint, Rustini Floranita, Gita Maya Koemara Sakti, Imran Pambudi, Lukas Hermawan, Benedicte Briot, Patricia Frenz, Orielle Solar, Pilar Campos, Eugenio Villar, Veronica Magar

**Affiliations:** a Gender, Equity and Human Rights, World Health Organization, Geneva, Switzerland; b Social Determinants of Health, World Health Organization, Geneva, Switzerland; c Gender, Equity and Human Rights, World Health Organization, Country Office for Indonesia, Jakarta, Republic of Indonesia; d Department of Maternal Health, Ministry of Health, Republic of Indonesia; e At the time of the Indonesia Innov8 pilot, he was with the Department of Maternal Health, Ministry of Health; f School of Public Health, University of Chile, Santiago, Chile; g Programme for Work, Employment, Equity and Health, Latin American Social Sciences Institute (FLACSO), Santiago, Chile; h Health Promotion Area, Ministry of Health, Social Services and Equality, Madrid, Spain

**Keywords:** Monitoring Health Inequality in Indonesia, Inequity, gender, determinants, human rights

## Abstract

The World Health Organization’s *Innov8 Approach for Reviewing National Health Programmes to Leave No One Behind* is an eight-step process that supports the operationalization of the Sustainable Development Goals’ commitment to ‘leave no one behind’. In 2014–2015, Innov8 was adapted and applied in Indonesia to review how the national neonatal and maternal health action plans could become more equity-oriented, rights-based and gender-responsive, and better address critical social determinants of health. The process was led by the Indonesian Ministry of Health, with the support of WHO. It involved a wide range of actors and aligned with/fed into the drafting of the maternal newborn health action plan and the implementation planning of the newborn action plan. Key activities included a sensitization meeting, diagnostic checklist, review workshop and in-country work by the review teams. This ‘methods forum’ article describes this adaptation and application process, the outcomes and lessons learnt. In conjunction with other sources, Innov8 findings and recommendations informed national and sub-national maternal and neonatal action plans and programming to strengthen a ‘leave no one behind’ approach. As follow-up during 2015–2017, components of the Innov8 methodology were integrated into district-level planning processes for maternal and newborn health, and Innov8 helped generate demand for health inequality monitoring and its use in planning. In Indonesia, Innov8 enhanced national capacity for equity-oriented, rights-based and gender-responsive approaches and addressing critical social determinants of health. Adaptation for the national planning context (e.g. decentralized structure) and linking with health inequality monitoring capacity building were important lessons learnt. The pilot of Innov8 in Indonesia suggests that this approach can help operationalize the SDGs’ commitment to leave no one behind, in particular in relation to influencing programming and monitoring and evaluation.

## Background

Around the world, Ministries of Health and others involved in the design, delivery, and monitoring and evaluation (M&E) of health programmes are grappling with the question of how to ‘leave no one behind’. This principle is reflected in the 2030 Agenda for Sustainable Development and is essential for the progressive achievement of universal health coverage (UHC) and realization of the right to health. The World Health Organization’s (WHO) *Innov8 Approach for Reviewing National Health Programmes to Leave No One Behind* aims to support these efforts at a concrete, programmatic level [1] as part of a wider UN-wide commitment to supporting operationalization of the leaving no one behind concept [2].

**Figure 1.**
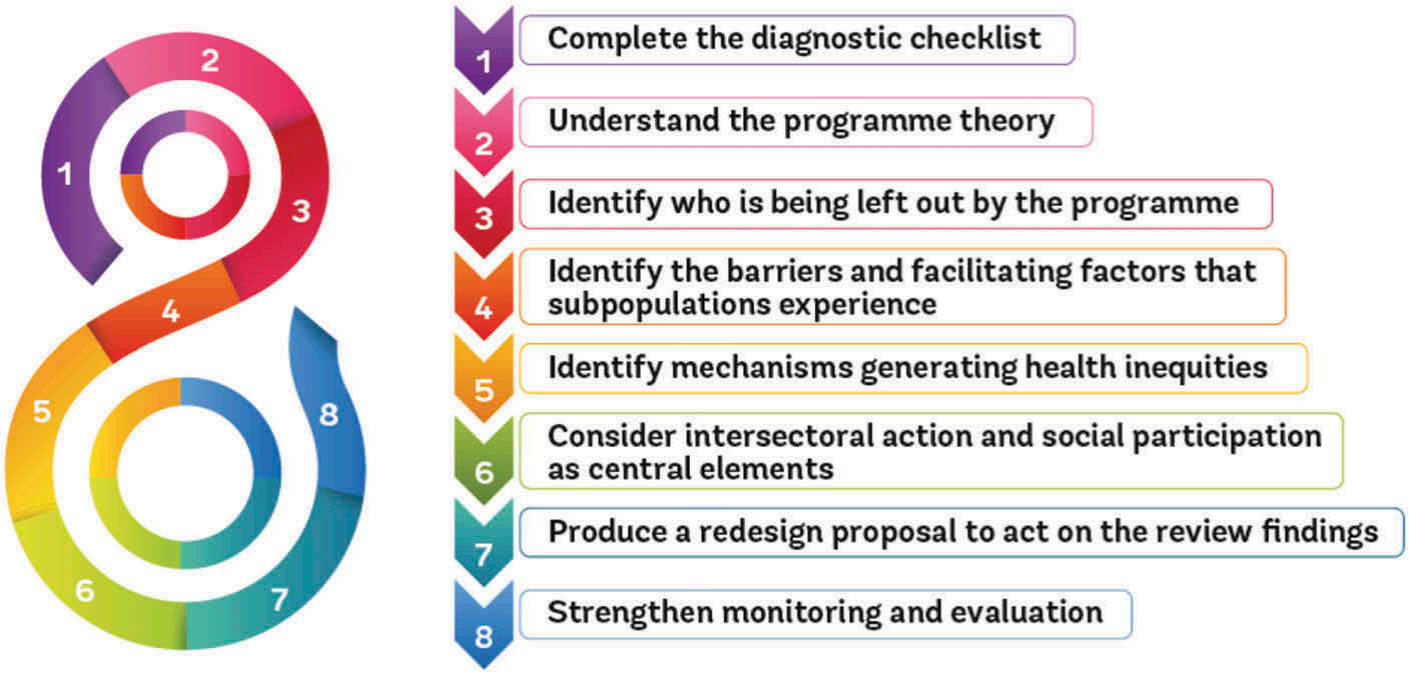
Eights steps of the *Innov8 Approach for Reviewing National Health Programmes to Leave No One Behind*. *Source:* WHO (2016) [1].

The Innov8 approach results in recommendations to strengthen the programme in order to make it more equity-oriented, rights-based and gender responsive, while addressing critical social determinants of health. Innov8 can be integrated to and/or aligned with ongoing programmatic review and planning cycles. The review process includes activities across the eight steps shown in Figure 1. The review is undertaken by a multidisciplinary team nominated by the Government, often comprising representatives from national and sub-national health authorities, research institutes, civil society and non-governmental organizations and other sectors.

The Innov8 process begins with the completion in **Step 1** of a diagnostic checklist and an articulation in **Step 2** about how the programme is expected to produce the desired results (a ‘programme theory’). Using available evidence, **Steps 3 and 4** respectively identify the sub-populations not reaching or benefiting less from the programme and the factors that prevent or hinder and facilitate effective coverage, serving to test the ‘programme theory’. In **Step 5**, the mechanisms underpinning these barriers and generating inequities and discrimination are examined. In **Step 6,** the review team considers how to overcome these barriers and challenges including through the enhancement of intersectoral action and social participation. A transformative redesign proposal is developed in **Step 7** that includes a set of action-oriented, targeted recommendations for adjustments to make the programme more equity-oriented, rights-based and gender-responsive and to address critical social determinants. **Step 8** looks at how to monitor the proposed programme enhancements and to adjust the ongoing M&E framework to ensure sustained attention to leaving no one behind.

The Innov8 approach has been carried out in the Americas, Eastern Mediterranean, European and South-East Asian regions of WHO. It can be adapted, tailored to and aligned with country-specific and programmatic contexts and existing review processes. It has been applied to programmes addressing sexual, reproductive, maternal, neonatal, child and adolescent health; non-communicable diseases; communicable diseases; and environmental health and health promotion, among others. It complements other WHO and UN resources (including those to support the *Global Strategy for Women’s, Children’s and Adolescents’ Health* [3] and the *Global Accelerated Action for the Health of Adolescents (AA-HA!)* [4]. Further information about the Innov8 approach *–* including about its evolution from where it began in Chile in 2008 to the present – can be found in the Innov8 technical handbook and on the WHO website [1,5].

This methods forum article describes the adaptation and application of the Innov8 methodology in Indonesia during 2014–2015, as well as outcomes, follow-up and select lessons learnt.

## Methods

In 2014 and 2015, the Ministry of Health (MOH), Government of Indonesia, co-organized with WHO a process to review how the national neonatal and maternal health (MNH) action plans could leave no one behind. The MOH led the review process. It worked closely with WHO staff from country, regional and global levels. Cooperation in this area was reflected in the Government of Indonesia and WHO *Country Cooperation Strategy (CCS) 2013–2017* [6]. CCS is a medium-term vision for WHO’s technical cooperation with a given Member State.

The rationale for applying Innov8 was the commitment of the MOH to reducing inequities in MNH, including those evidenced by the Demographic Health Survey 2012 [7]. These inequities are described in detail in other articles in this GHA series on ‘Monitoring Health Inequality in Indonesia’. In 2014, the Ministry leadership was aware of the need to tackle inequities in MNH, and wanted to reflect this in its planning approach. At that time, WHO shared information about Innov8’s use in other countries (for instance, sources [8] and [9]) for this purpose. The MOH and WHO agreed to pilot Innov8 to align with the drafting of its *Indonesia Maternal Health Action Plan 2016–2030* [10] and implementation planning for the *Indonesia Newborn Action Plan 2014* [11].

Acknowledging the importance of analysis across the continuum of care, the Innov8 review focused on essential MNH intervention packages for pre-pregnancy care; pregnancy care; childbirth and emergency obstetric and neonatal care; and newborn and postnatal care (see *A Global Review of the Key Interventions Related to Reproductive, Maternal, Newborn and Child Health* [12] for a generic description).

The overarching aims of the Indonesian review process are shown in Box 1.

The Innov8 review was overseen by the director of Maternal Health, MOH, and her directorate, supported by WHO. Approximately 50 people were engaged. They came from different parts of the MOH (i.e. the MNH programmes, the Centre for Data and Information, and the National Institute of Health Research and Development), select Provincial Health Offices, universities, research institutes, non-governmental organizations, the Global Health Partnership H6 (formerly H4+) and bilateral partners.

**Figure 2.**
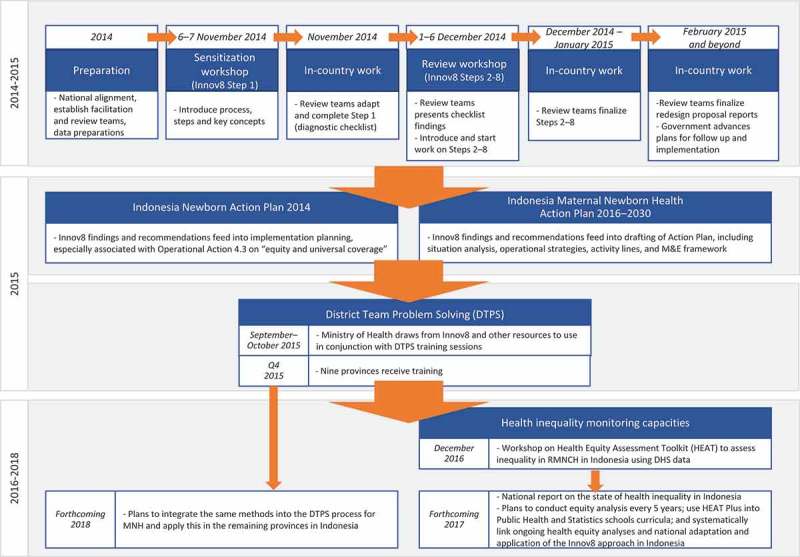
Overview of the Innov8 process in Indonesia and select follow-up activities.

The pilot involved a period of adaptation and preparation, a sensitization meeting in November 2014 followed by completion of the diagnostic checklist (Step 1), and a review meeting in December 2014 during which the other seven steps were introduced. This was followed by review team work during the December 2014 to April 2015 period to complete the steps. Figure 2 provides an overview of the process undertaken for the Innov8 review in Indonesia and select follow-up activities, the latter of which are explained in the next section.

## Results

The results of the Indonesia Innov8 review can be grouped in three categories:

- MNH action plans and operational planning;
- Equity-oriented planning integrated in the District Team Problem Solving (DTPS) approach;
- Generating demand for health inequality monitoring.

These are described in more detail below.

### MNH action plans and operational planning

Innov8 findings, among other sources, influenced the ‘leave no one behind’ focus in MNH action plans and operation planning [10,11]. For illustrative purposes, this section gives examples for the Maternal Health Action Plan, across its situation analysis, operational strategies (see the table in Appendix 1) and monitoring and evaluation framework.

The Innov8 review teams, through their analysis for sub-populations being left behind (from Step 3), had identified characteristics including the following as being important for disadvantage in relation to MNH: lower education, lower income, living in rural and remote areas, lacking identification, being an adolescent, being employed in the informal sector, and having a high-risk health condition (such as anaemia). Issues of marital status were also discussed, as was limited access to financial protection. Drawing from this, **situation analysis** of the Maternal Health Action Plan highlights health inequalities by age, sex, geographic area in the country, rural/urban residence, education, marital status and income.

Following on from the findings from the Innov8 Step 4 analysis on barriers, the Maternal Health Action Plan’s situation analysis also highlights a range of supply and demand-side barriers, including those linked to:

- insufficient numbers of adequately trained human resources;
- insufficient medicines and technologies at some community health facilities;
- inadequate/limited outreach capacity and counselling/information services in some locations;
- infrastructure shortages (such as inadequate electricity);
- transportation obstacles and extended time for travel;
- weak referral systems;
- low health literacy and weak health system navigation capacity;
- sociocultural factors (e.g. local beliefs on nutrition, disregard of antenatal services unless there is a problem, gender norms, and dismissive/discriminatory treatment by some providers).

The situation analysis also identifies barriers to financial protection, explaining that the Health Social Security (BPJS) programme is not always widely used by communities. The situation analysis further cites gender inequality issues, including the detrimental impacts of child marriages and adolescent pregnancies on girls’ physical and psychological well-being. These, and others, came forth through Innov8’s review of barriers using the Tanahashi framework for effective coverage [13] and then cross-analysis of these for the full continuum of care.

In terms of the Action Plan’s **operational strategies**, Innov8 findings (in particular for steps 5, 6 and 7) contributed to the focus in these on:

- Overcoming supply-side health system deficiencies influencing inequities in service coverage;
- Increasing the coverage with National Health Security, especially for the poor;
- Addressing barriers to services linked to geographical condition (e.g. isolated areas), insufficient transportation, sociocultural factors, and stigmatization of specific sub-populations (e.g. migrant groups in the cities);
- Activities on intersectoral action for maternal and child health (e.g. for infrastructure improvements linked to roads, communication and electrification; cooperation with religious sectors to increase the legal ages of marriage for men and women and provide information on reproductive health, among other topics);
- Activities on systematically involving the community in improving service quality (including community engagement approaches for the co-production of health and co-delivery of services).

Innov8 analysis for Step 8 informed the Action Plan’s **M&E framework**. This is reflected in the intermediate target calling for *reduction of inequalities in the coverage of maternal health services among and within the provinces to less than 10% based on education, economic status and place of residence stratifiers*. Multiple indicators in the framework also permit tracking of progress to overcome health system deficiencies causing inequities in the provision of quality services, which were also highlighted in Innov8 findings.

### Equity-oriented planning integrated in the district team problem solving (DTPS) approach

A challenge that emerged following the Innov8 application was that the review teams had produced many recommendations and they faced difficulties in knowing which should be prioritized, as some may be more relevant to parts of the country than others. The review teams concluded that prioritization of these was better done at sub-national level, given the decentralized context, variations in system capacity, and differing barriers to services across the country. The MOH decided to pilot integration of aspects of Innov8 into the District Team Problem Solving (DTPS) cycle. DTPS is undertaken by district teams to develop one-year proposals to address priority health problems facing the district [14].

During September–October 2015, the Ministry of Health worked to integrate a focus on leaving no one behind in select training sessions for district health authorities. Inputs drew on Innov8, and additional resources by WHO, UNICEF and partners on health inequality monitoring, qualitative assessment of barriers to services, and equity-oriented planning. Jogjakarta, Banten, West Java, Jakarta, East Nusa Tenggara, Aceh, Papua, West Papua and Central Java received this training in 2015. With this support and through follow-up work, districts and provinces included in their yearly proposals the identification of inequities and specific actions (and requests for resources) for narrowing the gaps.

After 2015, resource constraints, both human and financial, have hindered the further development and scaling up of this training approach. The Ministry of Health, with support from WHO and partners including academia, plan to review lessons learnt so far, conduct train-the-trainers activities in 2018, and then further advance efforts to integrate equity into the DTPS process for the remaining provinces.

### Generating demand for health inequality monitoring

Innov8 resulted in an increased demand for analysing, reporting, visualizing and disseminating data on health inequalities, as well as using that data in planning. In response, a MOH-WHO workshop was held in December 2016 to introduce the *Health Equity Assessment Toolkit* (HEAT), and help build national human resource capacity in this area. Follow-up actions include the development of a national report on the state of health inequality in Indonesia. Future plans include conducting equity analysis every five years using Indonesian data (as part of national SDGs monitoring), and integrating HEAT Plus (which allows users to upload and work with their own database) into Public Health and Statistics Schools’ curriculum. These activities are further described in the article by Hosseinpoor et al. [2018, 15] in this GHA series.

An opportunity and a challenge in the years to come will be to consistently integrate the data on health inequality into national and sub-national planning, monitoring and evaluation, and review activities. The DTPS-related trainings highlighted above will aid in this. The Ministry of Health and WHO also plan to further engage the Ministry of Planning in supporting this through joint activities in 2018.

## Conclusions

Conclusions regarding the Innov8 pilot in Indonesia focus on the issue of capacity, with regard to (1) enhanced capacity in the country; (2) building capacity across countries; (3) how the pilot contributed to improvement of the Innov8 methods to respond to country needs; and (4) building capacity for use of data on health inequality in planning and programming.

### Enhanced capacity in indonesia

Beyond the results featured in the above section, Innov8 has contributed to the capacities of the individuals, teams and institutions in Indonesia, as shown through the following examples:

- **Individuals**: Through Innov8 workshop evaluations, participants reported new knowledge relevant to leaving no one behind and abilities to apply this knowledge in their daily work. They reported that Innov8 had helped identify entry points for improving MNH-related programmes, including on areas where progress was perceived to have stagnated.
- **Teams**: Innov8, along with the *Global Accelerated Action for the Health of Adolescents (AA-HA!): Guidance to Support Country Implementation* [3], is being drawn from by MOH team members involved in the original innov8 training (and new staff) to review the draft National Action Plan on School Aged Children and Adolescent Health.
- **Institutions**: Innov8 raised awareness of service integration and wider health system strengthening issues that needed to be addressed for MNH services to have optimal and equitable impact. This awareness influenced the merging of the Ministry of Health’s maternal and child health directorates into a single Family Health Directorate. It also influenced related governance and strategic planning processes, aimed at fostering alignment.

### Building capacity across countries

WHO brokered exchange between Indonesia and other countries that had used Innov8. For instance, a representative from the Spanish Ministry of Health, Social Services and Equality was a co-facilitator of the sensitization workshop. WHO consultants from Chile (who had been engaged in the original design and oversight of the application in Chile) co-facilitated the review workshop. After the Indonesia pilot, a co-facilitator from Indonesia was involved in launching the Nepal Innov8 adaptation in 2015 [16]. A lesson learnt is that country-to-country exchange is important for conveying the rationale for reviewing programmes to leave no one behind, and describing processes for Innov8 adaptations and lessons learnt.

### The contribution of the Indonesia pilot to improving Innov8 methods

The Innov8 technical handbook, then still in draft form, and the accompanying workshop methods and materials were revised based on lessons from the Indonesia pilot. Changes included the reinforcement of gender and its intersections with other determinants in specific exercises, and a clearer articulation of how Innov8 supported operationalization of a human-rights-based approach to health. The sensitization meeting guidance was adjusted to provide deeper conceptual underpinnings related to equity, gender, human rights and social determinants. The guidance for a third workshop to support Steps 7 and 8 (which happened spontaneously in Indonesia amongst the review team leads) was consolidated and became a formal part of the Innov8 process guidance.

### Building capacity for use of data on health inequality in planning and programming

National authorities in Indonesia found it useful to draw from Innov8 and *Health Equity Assessment Toolkit* (HEAT) together. Applying them together generates a ‘virtuous cycle’ in which Innov8 enables the identification of ways to act on and address the identified health inequalities. It strengthens the systematic use of health inequality data to support programme planning cycles. Meanwhile, HEAT enables data on health inequalities to be analysed.

The Innov8 application in Indonesia is an example of efforts being made – across country contexts – to close the critical gap that currently exists between the commitment to ‘leaving no one behind’ and the capacity to progressively realize this commitment through concrete, operational means in planning and programming cycles. Continued learning and sharing of knowledge from such efforts will be central as progress advances towards the SDGs.

## Paper context

The Global Health Action series ‘Monitoring Health Inequality in Indonesia’ highlights inequalities that are important to tackle on the path to universal health coverage. This article provides an example of how the Government of Indonesia and WHO used the *Innov8 approach to reviewing national health programmes to leave no one behind* to integrate a focus on tackling inequities into the national neonatal and maternal health action plans. It describes lessons learnt and next steps.

## Appendix 1. The operational strategies framework of the 2016–2030 Maternal Health Action Plan of the Republic of Indonesia

**Table 1.** The operational strategies framework of the 2016–2030 Maternal Health Action Plan of the Republic of Indonesia.

Operational strategy	Activities	Targets by 2030
Strategy 1. Achieving universal coverage with maternal and neonatal health services and overcoming inequities in service coverage	Ensuring adequate logistics/supplies for basic and referral maternal and neonatal health services; Reducing inequities in coverage of maternal and neonatal health services.	95% of the total districts have the capability to ensure logistics/supplies for basic and referral maternal and neonatal health services; 100% of the total districts provide maternal and neonatal health services for persons living in isolated villages; 100% of subpopulations experiencing inequities/vulnerable populations are covered with the National Health Security.
Strategy 2. Improving the quality of maternal health services and referral services	Improving the quality of antenatal services, including by ensuring comprehensive and integrated services; Improving the quality of obstetric services by implementing obstetric care standards for normal childbirth; Improving the quality of emergency obstetric/neonatal services in basic health services (PONED) and at the referral level in district hospitals (PONEK); Establishing maternal-perinatal services networks/ regions by involving hospitals and private practices.	95% of the total health facilities implement comprehensive and integrated antenatal service standards; 95% of the total health facilities use a partograph to monitor obstetric progress; 100% of the total districts/municipalities have 1 hospital that is able to provide basic health services per 500,000 population; 100% of the total districts/ municipalities own 4 community health centers that are able to provide basic health services; 100% of the total provinces have effective maternal-perinatal service networks/ minimum of 4 regional hospitals per province providing care following referral from district hospitals.
Strategy 3. Establishing sustainable and integrated maternal health services	Maintaining sustainable maternal and neonatal health services.	100% of the total districts with less than 10% inequality in the coverage of maternal and neonatal health services.
Strategy 4. Developing partnerships with associated sectors/parties and encouraging active community participation	Improving partnerships with associated sectors and parties; Involving community participation in improving maternal and neonatal health quality.	100% of the districts/municipalities support intersectoral action for maternal and child health; 100% of the total provinces have a Health Board; 90% of the total districts involve the community systematically in improving maternal and neonatal health quality.
Strategy 5. Strengthening leadership in management of the maternal health programme, including through the improvement of transparency and accountability.	Increasing professionalism in managing the Maternal Health Programme and maternal and neonatal health services; Improving subnational independence in achieving quality universal maternal and neonatal health services.	100% of the districts have the competency in managing effectively and efficiently the Maternal Health Programme. 100% of the districts/municipalities allocate more than 10% of the local government budget for the health sector.

Source: Ministry of Health of the Republic of Indonesia (2015) [9].

**Box 1.** Aims of the Innov8 review of the MNH action plans.

To jointly review data on inequities in maternal and neonatal health – by wealth quintile, region/province, rural/urban, age, education of mother, sex of child, and/or other relevant stratifiers;
To jointly consider ‘what worked and what did not’ with regard to how previous MNH services performed in relation to those inequities; To identify key entry points for strengthening the equity-oriented, rights-based, gender-responsive and social determinants focus in MNH services; To propose options for enhancing the ‘leave no one behind focus’ in monitoring and evaluation approaches to MNH; and To build the capacity of health professionals to apply related concepts and underlying principles in their ongoing work.
